# Tigecycline Susceptibility and the Role of Efflux Pumps in Tigecycline Resistance in KPC-Producing *Klebsiella pneumoniae*

**DOI:** 10.1371/journal.pone.0119064

**Published:** 2015-03-03

**Authors:** Fang He, Ying Fu, Qiong Chen, Zhi Ruan, Xiaoting Hua, Hua Zhou, Yunsong Yu

**Affiliations:** 1 Department of Infectious Diseases, Sir Run Run Shaw Hospital, School of Medicine, Zhejiang University, Hangzhou, Zhejiang, 310016, China; 2 Department of Respiratory Diseases, The First Affiliated Hospital, School of Medicine, Zhejiang University, Hangzhou, Zhejiang, 310003, China; Quuen's University Belfast, UNITED KINGDOM

## Abstract

KPC-producing *Klebsiella pneumoniae* isolates have emerged as important pathogens of nosocomial infections, and tigecycline is one of the antibiotics recommended for severe infections caused by KPC-producing *K*. *pneumoniae*. To identify the susceptibility profile of KPC-producing *K*. *pneumoniae* to tigecycline and investigate the role of efflux pumps in tigecycline resistance, a total of 215 KPC-producing *K*. *pneumoniae* isolates were collected. The minimum inhibitory concentration (MIC) of tigecycline was determined by standard broth microdilution tests. Isolates showing resistance to tigecycline underwent susceptibility test with efflux pump inhibitors. Expression levels of efflux pump genes (*acrB* and *oqxB*) and their regulators (*ramA*, *marA*, *soxS* and *rarA*) were examined by real-time PCR, and the correlation between tigecycline MICs and gene expression levels were analysed. Our results show that the tigecycline resistance rate in these isolates was 11.2%. Exposure of the tigecycline-resistant isolates to the efflux pump inhibitor NMP resulted in an obvious decrease in MICs and restored susceptibility to tigecycline in 91.7% of the isolates. A statistically significant association between *acrB* expression and tigecycline MICs was observed, and overexpression of *ramA* was found in three tigecycline-resistant isolates, further analysis confirmed *ramR *mutations existed in these isolates. Transformation of one mutant with wild-type *ramR* restored susceptibility to tigecycline and repressed overexpression of *ramA* and *acrB*. These data indicate that efflux pump AcrAB, which can be up-regulated by *ramR* mutations and subsequent *ramA* activation, contributed to tigecycline resistance in *K*. *pneumoniae* clinical isolates.

## Introduction

*Klebsiella pneumoniae* has emerged worldwide as an important pathogen of nosocomial infections that causes a variety of infections, including pneumonia, liver abscesses, urinary-tract infections and bacteraemia. Carbapenems are often the last resort for treating infections due to the emergence of multidrug-resistant *K*. *pneumoniae* [1]. However, the acquisition of carbapenemase has contributed to resistance to all β-lactams including carbapenem antibiotics. Carbapenem-hydrolysing *Klebsiella pneumoniae* carbapenemase (KPC)-type enzymes have been identified mostly in *K*. *pneumoniae*. In fact, most KPC carbapenemase-producing *K*. *pneumoniae* show resistance to almost all antibiotics except colistin and tigecycline.

Tigecycline, one type of glycylcycline, is a novel expanded-spectrum antibiotic. It is a derivative of minocycline, which inhibits the initial codon recognition step of tRNA accommodation and prevents rescue by the tetracycline resistance protein TetM [2, 3]. Tigecycline is effective against most carbapenemase-producing bacteria including *K*. *pneumoniae* and has been approved for clinical use in China during recent years. *K*. *pneumoniae* has previously been reported to be non-susceptible to tigecycline in other countries [4, 5]. The resistance rate to tigecycline in multidrug-resistant *K*. *pneumoniae* in the USA was approximately 9.2% (MIC≥8 mg/L, FDA) [6], while the resistance rate in ESBL-producing isolates was approximately 33.3% in Spain (MIC >2 mg/L, EUCAST) [7].

The mechanism of tigecycline resistance has not yet been clearly elucidated. It has been reported that the increased expression of efflux pumps such as AcrAB and OqxAB is one of the possible mechanisms [4, 8, 9]. Expression of the *acrAB* operon is controlled by its local repressor AcrR [10]. Several global transcriptional regulators of the AraC family, RamA, MarA, SoxS, and RarA, may participate in tigecycline resistance via efflux pump activation [5, 11, 12]. *ramR*, *marR* and *soxR* are repressors of *ramA*, *marA* and *soxS*, respectively. RamA is also regulated by the Lon protease [13]. Mutation in these genes might be responsible for *ramA*, *marA* and *soxS* overexpression that subsequently leads to upregulation of the efflux pumps [14–16].

In This study, a total of 215 KPC-producing *K*. *pneumoniae* were collected from four hospitals in three provinces in China. We identified the tigecycline susceptibility profiles of these isolates. Furthermore, we investigated the role of efflux pumps and the function of regulators in tigecycline resistance.

## Material and Methods

### Bacterial isolates

A total of 215 KPC-producing *K*. *pneumoniae* isolates were collected between Jan. 2010 and Dec. 2013 from the following centres in China: First Affiliated Hospital, School of Medicine, Zhejiang University (ZJF); Sir Run Run Shaw Hospital, School of Medicine, Zhejiang University (ZJS); The First Affiliated Hospital of Kunming Medical University (KM); The First Affiliated Hospital of Zhengzhou University (ZZ). All isolates were identified using the VITEK 2 system (bioMérieux, France). The *bla*_KPC_ gene was amplified to confirm the KPC-producing *K*. *pneumoniae* isolates [17].

### Antimicrobial susceptibility test

The MIC of tigecycline was determined using standard broth microdilution tests with fresh (<12 h) ISO-Sensitest broth (Oxoid LTD, Basingstoke, Hampshire, England). MIC results were interpreted according to the European Committee on Antimicrobial Susceptibility Testing (EUCAST) clinical breakpoints (for tigecycline, ≤1.0 mg/L is susceptible, 2.0 mg/L is intermediate, and >2.0 mg/L is resistant) [18]. *Escherichia coli* ATCC 25922 was used for quality control in the susceptibility assays. Isolates that showed resistance to tigecycline also underwent susceptibility testing for tigecycline by adding efflux pump inhibitors 1-(1-naphthylmethyl)-piperazine (NMP), phenylalanine arginine β-naphthylamide (PAβN) or carbonyl cyanide m-chlorophenylhydrazone (CCCP) to the medium [19]. The MICs of other antimicrobial agents were determined using the agar dilution method or Etest method.

### PFGE analysis

Genomic DNA was digested with restriction enzyme XbaI (TaKaRa, Dalian, China), and DNA fragments were separated by electrophoresis in 1% agarose III (Sangon, Shanghai, China) in 0.5× TBE (45 mM Tris, 45 mM boric acid, 1.0 mM EDTA; pH 8.0) buffer with a CHEF apparatus (CHEF Mapper XA, Bio-Rad, USA) at 14°C and 6 V/cm and with alternating pulses at a 120° angle in a 6 to 36 s pulse time gradient for 22 h. The results of PFGE were analysed using BioNumerics 7.0 (Applied Maths, Austin, TX, USA) software.

### Real-time PCR

mRNA expression levels of efflux pump genes (*acrB* and *oqxB*) and regulators (*ramA*, *marA*, *soxS* and *rarA*) were examined by real-time PCR. Overnight bacterial cultures were diluted 1/100 in LB broth (Sangon, Shanghai, China) and grown to log phase at 37°C with vigorous shaking (230 rpm). RNase-free DNase (TaKaRa, Dalian, China)-treated RNA was harvested using the Purelink RNA Mini Kit (Ambion, Carlsbad, USA). The yield and quality of RNA were determined using a Nanodrop 2000C (Thermo, USA). Two micrograms of total RNA were reverse transcribed into cDNA using the PrimeScript RT Reagent kit (TaKaRa, Dalian, China). Real-time quantitative RT-PCR was run on a LightCycler 480 II (Roche, Germany) with 40 cycles of 5 s at 95°C, 30 s at 54°C, and 30 s at 72°C. SYBR Premix Ex Taq (TaKaRa, Dalian, China) was used to quantify the expression of the target gene. The reactions were performed in a volume of 20 μL. All experiments were performed in triplicate. The primers used in these experiments are listed in Table 1. Expression of each gene was normalised to that of a housekeeping gene (*rpoB*). Relative expression of each target gene was then calibrated against the corresponding expression of a tigecycline-susceptible isolate K134 (expression = 1), which served as the control. Data were analysed by using the 2^-ΔΔCT^ method.

**Table 1:** Primers used for real-time PCR studies and PCR amplification.

Primers for this study (5’-3’)			Usage	Reference
*rpoB*	*rpoB*-F	CCGTATCTACGCTGTGCT	RT-PCR	This study
	*rpoB*-R	TGTTACCGTGACGACCTG	RT-PCR	This study
*acrB*	*acrB*-F	CGATAACCTGATGTACATGTCC	RT-PCR	[27]
	*acrB*-R	CCGACAACCATCAGGAAGCT	RT-PCR	[27]
*oqxB*	*oqxB*-F	CGAAGAAAGACCTCCCTACCC	RT-PCR	This study
	*oqxB*-R	CGCCGCCAATGAGATACA	RT-PCR	This study
*ramA*	*ramA*-F	GCATCAACCGCTGCGTATT	RT-PCR	This study
	*ramA*-R	GGGTAAAGGTCTGTTGCGAAT	RT-PCR	This study
*marA*	*marA*-F	TAATGACGCCATCACTATCCA	RT-PCR	This study
	*marA*-R	ATGTACTGGCCGAGGGAATG	RT-PCR	This study
*soxS*	*soxS*-F	TAGTCGCCAGAAAGTCAGGAT	RT-PCR	This study
	*soxS*-R	AGAAGGTTTGCTGCGAGACG	RT-PCR	This study
*rarA*	*rarA*-F	GTTTGTTGACGAAGTGCA	RT-PCR	This study
	*rarA*-R	GCCATCATTTCCAGGGTA	RT-PCR	This study
*ramR*	*ramR*-F	GATGGCGACCACGCTAAA	Amplification	This study
	*ramR*-R	GCTCGGTAAACGGGTAGGT	Amplification	This study
*lon*	*lon*-F	TCCCGCCGTTGAATGTGTGG	Amplification	This study
	*lon*-R	ACTTACCAGCCCTATTTTTAT	Amplification	This study
*marR*	*MarR*-F	TAATGTTGACTTATGATTGCCT	Amplification	This study
	*MarR*-R	ACATCATCTTACCTCTTCTT	Amplification	This study
*soxR*	*SoxR*-F	TTTTGTCTGCGGGCGAGTAT	Amplification	This study
	*SoxR*-R	GCGAGATAATGCGAAAGACA	Amplification	This study

### Statistical analysis

The association between MICs and gene expression levels was analysed using the SPSS Statistics 17.0 software. According to the normality test and homogeneity of variances test, expression levels of *soxS*, *marA*, *rarA* and *oqxB* appear to be a normal distribution with equal variance, so an analysis of variance (ANOVA) statistical test was adopted. The expression levels of *acrB* and *ramA* appear to be a normal distribution with unequal variance, so a Kruskal-Wallis Test was adopted. Statistical significance was established by using a conventional level of *P <* 0.05.

### Mutation analysis of *acrR*, *ramR*, *marR*, *soxR* and *lon*

*acrR*, *ramR*, *marR*, *soxR* and *lon*, were amplified and sequenced to identify mutations within these genes. The primers designed for these studies are listed in Table 1.

### *ramR* plasmid construction and transformation

A DNA fragment carrying the wild-type *ramR* gene was amplified from a tigecycline-susceptible isolate (K134) with the primers listed in Table 1. After amplification, the amplimer was cloned into pCR-Blunt II -TOPO. The mutant strain S21 (kanamycin-susceptible) was used for transformation. The influence of the *ramR* mutation on the tigecycline MIC and transcriptional expression levels of *ramA* and *acrB* was examined using standard broth microdilution tests and real-time RT-PCR.

## Results

### Tigecycline resistance and MICs distribution

**Fig 1:**
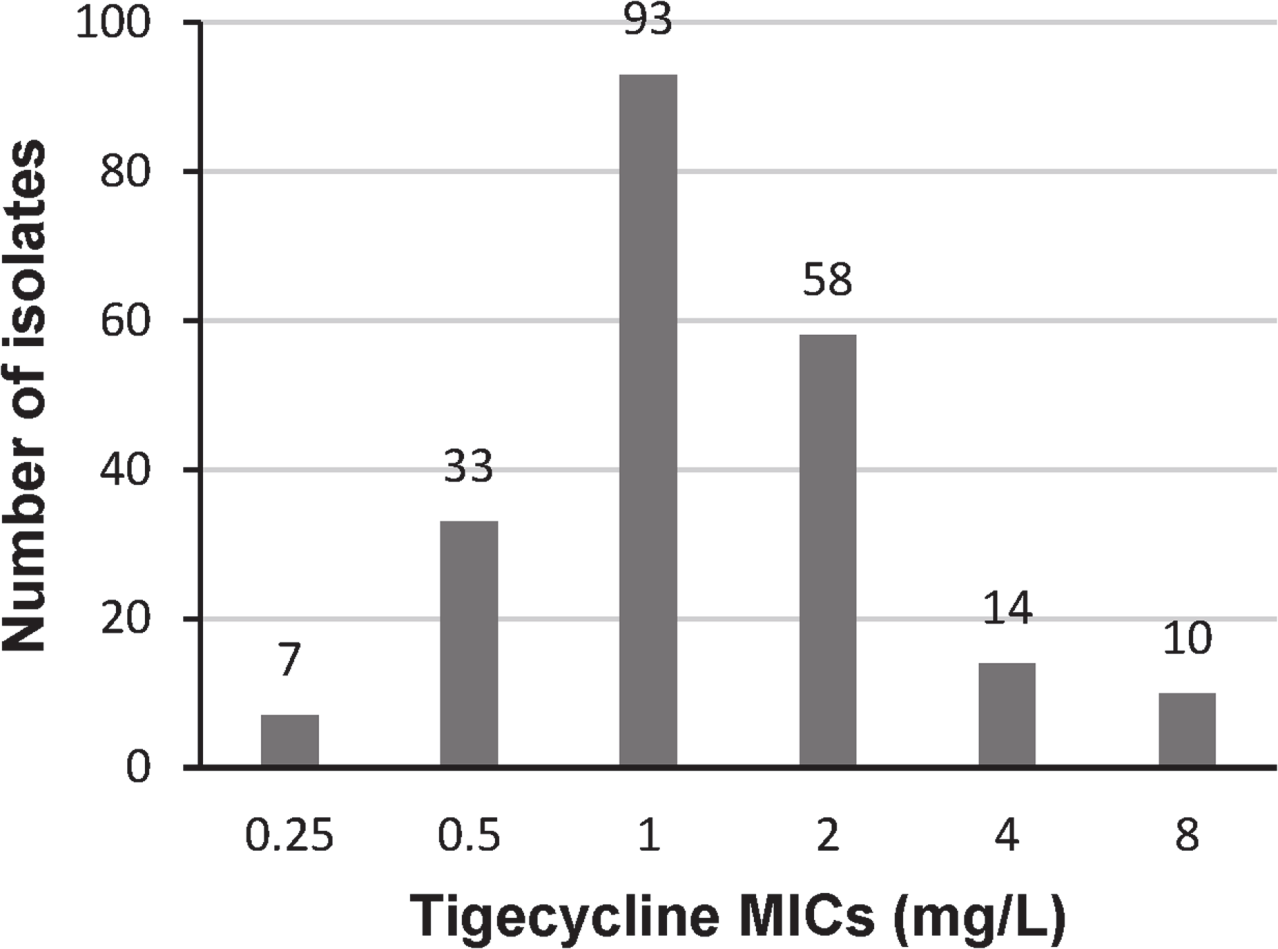
Tigecycline MIC distribution for 215 KPC-producing *K*. *pneumoniae* clinical isolates.

Of the 215 KPC-producing *K*. *pneumoniae* isolates, 24 isolates were resistant to tigecycline (MIC>2 mg/L). The MIC distribution is presented in Fig. 1. The tigecycline resistance rate for these strains was 11.2% (MIC>2 mg/L, EUCAST). The range of tigecycline MICs was 0.25–8 mg/L. The MIC_50_ and the MIC_90_ were 1 and 4 mg/L, respectively.

The results of antimicrobial susceptibility testing of tigecycline-resistant isolates are presented in Table 2. All isolates were resistant to multiple antimicrobial agents. Exposure of tigecycline-resistant isolates to the efflux pump inhibitor NMP resulted in an obvious decrease (4 to 16-fold) in the MICs of tigecycline and restored susceptibility for all except two strains (S21 and K23), while susceptible isolate MICs declined slightly from 0.75 to 0.125 mg/L. The effects of PAβN and CCCP were not significant (Table 2).

**Table 2:** Susceptibilities of 24 tigecycline-resistant isolates to 12 antimicrobial agents and MIC values of tigecycline in the presence of efflux pump inhibitors NMP, PAβN or CCCP.

Isolate	Clonal group	Hospital	MIC (mg/L) ^a^														
			TZP^b^	CAZ^b^	FEP^b^	SCF^b^	IPM^b^	MEM^b^	AK^c^	CIP^c^	TS^c^	MC^c^	CO^c^	TGC	TGC+NMP	TGC+PAβN	TGC+CCCP
K22	A	ZJF	>256	128	64	>256	32	16	>256	>32	>32	8	0.25	4	0.25	1	1
K23	B	ZJF	256	>256	32	64	16	8	>256	>32	>32	32	0.25	8	4	2	8
K83	C	ZJF	>256	128	128	>256	128	64	0.25	>32	>32	32	0.5	4	1	4	2
Y13	D	ZJF	>256	>256	>256	>256	128	256	>256	>32	0.25	6	0.5	4	1	2	4
Y17	E	ZJF	>256	>256	>256	>256	256	>256	>256	>32	1	8	0.5	8	1	2	4
S21	F	ZJS	>256	256	64	>256	8	128	1.5	4	>32	>256	0.5	8	2	4	8
H65	G	KM	>256	256	32	>256	64	128	>256	>32	1	8	0.5	8	1	2	8
Q4	H	ZZ	>256	>256	64	>256	64	256	>256	>32	>32	4	0.5	4	1	2	4
Q5	H	ZZ	>256	>256	256	>256	64	128	>256	>32	>32	4	0.5	4	1	2	4
Q6	H	ZZ	>256	>256	256	>256	64	64	>256	>32	>32	4	0.5	4	0.5	4	2
Q8	H	ZZ	>256	>256	256	>256	64	128	>256	>32	>32	8	0.5	4	1	2	2
Q10	H	ZZ	256	>256	256	>256	64	128	>256	>32	>32	8	0.5	4	1	4	2
Q11	H	ZZ	>256	>256	256	>256	64	128	>256	>32	>32	4	0.5	4	0.5	2	2
Q12	H	ZZ	>256	>256	256	>256	64	64	>256	>32	>32	8	0.5	8	0.5	4	4
Q14	H	ZZ	>256	>256	256	>256	64	128	>256	>32	>32	8	0.5	8	0.5	4	4
Q15	H	ZZ	>256	>256	256	>256	64	64	>256	>32	>32	4	0.5	4	0.5	4	2
Q17	H	ZZ	>256	>256	>256	>256	64	>256	0.5	>32	>32	4	0.5	4	0.5	4	2
Q20	H	ZZ	>256	>256	256	256	64	128	>256	>32	>32	4	0.5	8	1	4	4
Q22	H	ZZ	>256	>256	256	>256	128	256	>256	>32	>32	4	0.5	4	0.5	2	2
Q28	I	ZZ	>256	64	256	>256	128	256	1	>32	>32	8	0.5	8	0.5	4	2
Q30	H	ZZ	>256	>256	64	>256	128	256	1	>32	>32	4	0.5	4	0.5	4	4
Q38	H	ZZ	>256	>256	>256	>256	128	128	>256	>32	>32	4	0.5	8	0.5	4	4
Q39	H	ZZ	>256	>256	>256	>256	128	256	>256	>32	>32	4	0.5	8	0.5	4	4
Q40	H	ZZ	>256	>256	>256	>256	128	128	0.5	>32	>32	4	0.5	4	0.5	2	4

^a^Abbreviations: TZP, piperacillin/tazobatam; CAZ, ceftazidime; FEP, cefepime; SCF, cefoperazone/sulbactam; IPM, imipenem; MEM, meropenem; AK, amikacin; CIP, ciprofloxacin; TS, trimethoprim/sulfamethoxazole; MC, minocycline; CO, colistin; TGC, tigecycline; TGC+NMP, tigecycline with NMP; TGC+PAβN, tigecycline with PAβN, TGC+CCCP, tigecycline with CCCP

^b^Tested by agar dilution method

^c^Tested by Etest method

### PFGE analysis

**Fig 2:**
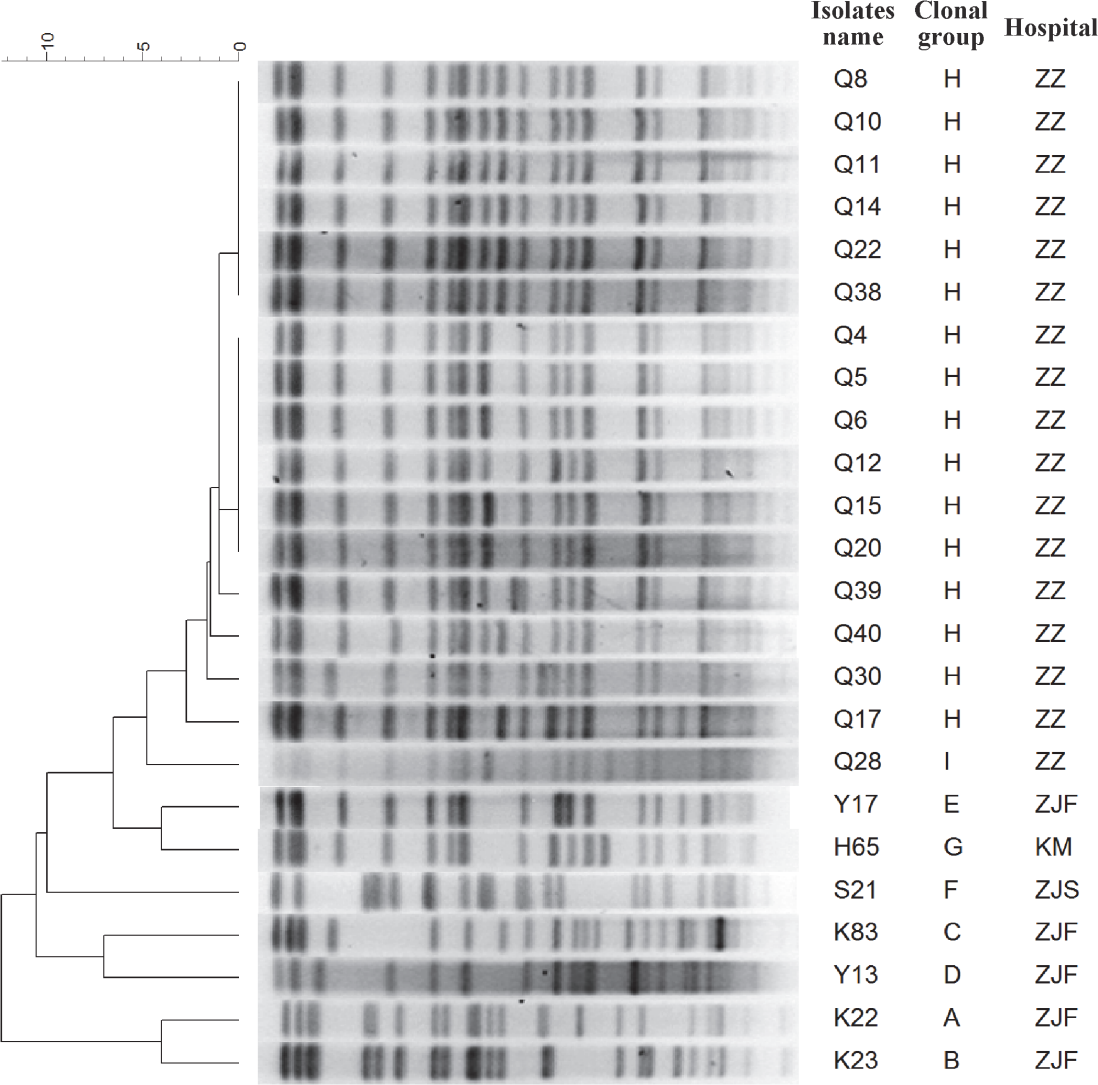
Phylogenetic clone analysis of 24 tigecycline-resistant isolates . These isolates were divided into 9 clonal groups (group A to I).

Three groups of isolates were selected for PFGE analysis; the 24 tigecycline-resistant isolates were designated as group one, a random selection of 24 isolates (equal to the number of tigecycline-resistant isolates) with a MIC = 1 mg/L isolates were designated as group two, and seven total isolates with a MIC = 0.25 mg/L were designated as group three. PFGE analysis revealed that isolates in group one could be divided into nine clonal groups (Fig. 2), group two isolates could be divided into 13 clonal groups (S1 Fig.), and group three isolates could be divided into seven clonal groups (S2 Fig.).

Among the tigecycline-resistant isolates, all five isolates from ZJF hospital are clonally distinct (group A—E). One isolate from ZJS hospital belongs to an individual clonal group (group F), and the isolate from KM hospital also belongs to an individual clonal group (group G). Clone dissemination was observed in ZZ hospital isolates. Sixteen of 17 isolates were clustered together with similar patterns belonging to clone group H, and the other isolate belonged to an individual clonal group (group I).

### Gene expression analysis and relationship with tigecycline MICs

**Fig 3:**
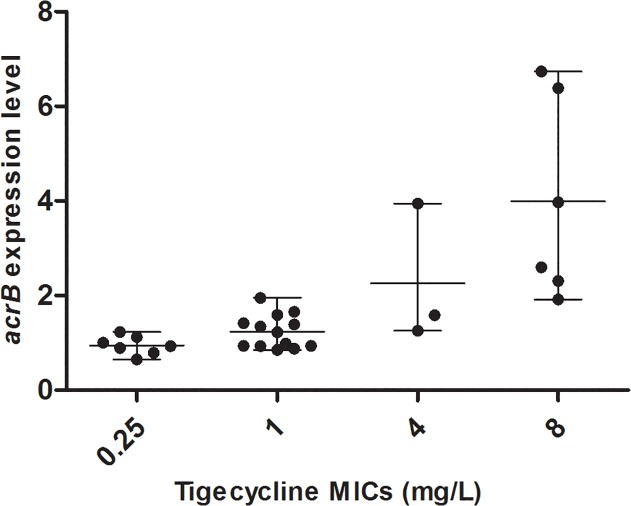
Relationship between *acrB* expression levels and tigecycline MICs.

Clonally distinct isolates from each MIC group were selected for gene expression analysis. The gene expression levels of different MIC groups are presented in Table 3. According to the results of the Kruskal-Wallis Test (Table 4), there was a statistically significant association between *acrB* expression and tigecycline MICs (*P*<0.05). The Kruskal-Wallis Test indicates that *acrB* expression level in isolates with MICs of ≥4 mg/L is statistically significantly different from those with MICs of 1 mg/L and 0.25 mg/L and that *acrB* expression in isolates with MICs of 1 mg/L is also statistically significantly different from MICs of 0.25 mg/L. From these results, we found a trend for higher *acrB* expression as the tigecycline MIC increases (Fig. 3).

**Table 3:** Expression of *acrB*, *ramA*, *soxS*, *marA*, *rarA*, *oqxB* and mutation of *acrR*, *ramR* in different MIC groups of *K*. *pneumoniae* clinical isolates.

Isolate	MIC(mg/L) ^c^	Relative expression ^a^						Mutation	
		*acrB*	*ramA*	*soxS*	*marA*	*rarA*	*oqxB*	*acrR*	*ramR*
K134	0.25	1	1	1	1	1	1	-	-
K27	0.25	0.93±0.09	1.56±0.66	2.00±1.08	4.50±1.36	1.65±0.56	2.04±1.20	-	-
K32	0.25	0.89±0.16	1.53±0.42	1.83±0.86	5.05±1.15	1.58±1.02	2.29±1.18	-	-
K72	0.25	1.12±0.23	0.97±0.37	1.65±0.37	3.28±1.80	1.58±0.51	2.47±0.97	-	-
K82	0.25	0.79±0.07	1.00±0.08	1.13±0.33	3.43±0.69	1.05±0.14	1.23±0.34	-	-
K135	0.25	0.65±0.12	0.71±0.27	0.90±0.44	2.79±0.46	0.75±0.22	0.73±0.33	-	-
K148	0.25	1.23±0.10	ND^b^	1.92±0.76	5.62±1.66	1.26±0.16	3.55±2.89	-	-
K16	1	0.98±0.39	1.33±0.60	1.70±0.76	6.01±3.39	0.96±0.26	1.77±0.76	-	-
K25	1	0.88±0.18	1.77±0.94	1.64±1.02	4.98±4.09	0.92±0.55	1.63±1.10	-	-
K29	1	0.94±0.19	1.06±0.50	1.36±0.74	4.21±1.87	1.28±0.92	1.54±1.10	-	-
K49	1	0.85±0.19	0.73±0.41	0.79±0.50	2.64±1.38	0.62±0.23	ND^b^	-	-
K76	1	0.93±0.17	1.49±0.49	1.74±0.58	5.51±1.95	1.30±0.33	2.50±0.19	-	-
K101	1	1.42±0.21	0.98±0.51	1.09±0.67	3.38±1.87	0.98±0.43	1.17±0.56	IS5^d^	-
K128	1	0.94±0.28	ND^b^	1.52±0.95	4.99±3.83	0.98±0.28	1.68±0.80	-	-
K155	1	1.22±0.34	0.96±0.57	1.12±0.59	3.93±2.33	0.68±0.27	ND^b^	IS5^d^	-
Y8	1	1.39±0.25	ND^b^	1.50±1.31	4.62±4.25	0.98±0.52	ND^b^	IS5^d^	-
H33	1	1.34±0.42	1.14±0.68	1.34±0.72	4.23±2.51	0.87±0.42	ND^b^	IS5^d^	-
S7	1	1.95±0.15	2.07±0.18	2.33±0.11	6.86±0.84	1.51±0.03	2.91±0.34	IS5^d^	-
S10	1	1.66±0.16	1.38±0.41	1.53±0.50	4.39±1.96	1.30±0.20	ND^b^	IS5^d^	-
S17	1	1.59±0.18	ND^b^	1.00±0.42	3.24±1.43	0.95±0.22	ND^b^	IS5^d^	-
K22	≥4	1.26±0.21	1.10±0.39	0.99±0.43	3.54±1.11	0.76±0.11	1.00±0.28	A20D	-
K23	≥4	1.92±0.40	0.29±0.12	0.17±0.06	1.02±0.13	0.37±0.11	3.04±0.87	A20D	-
K83	≥4	1.58±0.15	2.71±0.41	2.24±0.64	11.48±1.76	1.56±0.35	3.35±0.29	-	-
Y13	≥4	3.94±0.30	1.93±0.50	1.02±0.21	3.89±0.98	0.82±0.07	ND^b^	IS5^d^	-
Y17	≥4	6.38±2.64	9.43±3.89	0.67±0.48	2.11±1.08	0.70±0.22	1.24±0.48	IS5^d^	E113K
H65	≥4	6.74±1.06	7.82±2.17	1.17±0.24	4.49±1.76	1.06±0.08	ND^b^	IS5^d^	I106F
S21	≥4	3.97±0.49	13.77±2.90	1.94±0.74	7.19±2.25	1.88±0.71	2.11±0.94	-	Q122Stop
Q28	≥4	2.31±0.28	0.73±0.08	1.01±0.22	2.30±0.68	0.67±0.09	ND^b^	IS5^d^	-
Q38	≥4	2.60±0.43	1.67±0.42	1.84±0.66	5.27±1.67	1.79±1.17	2.84±1.73	IS5^d^	-

^a^ Relative expression compared with K134 (expression = 1). Results are means of 3 runs ± standard deviation.

^b^ ND, not determined. Mutations in primer region or gene deletion may have affected expression.

^c^ MIC of tigecycline.

^d^ IS*5* insertion element into the nucleotide position 276–277 of the *acrR* gene.

**Table 4:** Kruskal-Wallis Test of *acrB* and *ramA* expression on the tigecycline MICs.

Gene	Number of clonally distinct isolates	MIC	*x̅*±*s*	χ^2^	*P*
*acrB*	7	0.25	0.94±0.20	16.201	0.001
	13	1	1.24±0.35		
	9	≥4	3.41±2.02		
*ramA*	6^a^	0.25	1.13±0.34	3.345	0.188
	10^b^	1	1.29±0.41		
	9	≥4	4.38±4.78		

^a^ One isolate was not determined.

^b^ Three isolate were not determined.

The relationship between *oqxB* expression and tigecycline MICs was not evident (Table 5). High expression of *oqxB* was found both in tigecycline resistant and susceptible isolates (Table 3). The role of OqxAB pump in tigecycline resistance was uncertain.

**Table 5:** ANOVA of *marA*, *soxS*, *rarA* and *oqxB* expression on the tigecycline MICs.

Gene	Number of clonally distinct isolates^a^	MIC	*x̅*±*s*	*F*	*P*
*marA*	7	0.25	3.67±1.56	0.495	0.615
	13	1	4.54±1.16		
	9	≥4	4.59±3.17		
*soxS*	7	0.25	1.49±0.47	0.650	0.530
	13	1	1.44±0.39		
	9	≥4	1.23±0.66		
*rarA*	7	0.25	1.27±0.35	0.930	0.407
	13	1	1.03±0.26		
	9	≥4	1.07±0.54		
*oqxB*	7	0.25	1.90±0.99	0.382	0.688
	7 ^a^	1	1.89±0.60		
	6 ^b^	≥4	2.26±0.98		

^a^ Five isolates were not determined.

^b^ Three isolates were not determined.

Overexpression of *ramA* were found in tigecycline resistant isolates (Y17, H65, S21) which also with high expression level of *acrB* (Table 3). Overexpression of *ramA* could contribute to the up-regulation of *acrB* in these isolates. The difference of *marA*, *soxS*, and *rarA* expression between tigecycline resistant isolates and susceptible isolates were not significant.

### Mutation analysis of *acrR*, *ramR*, *marR*, *soxR* and *lon*

The sequences of the *lon*, *marR* and *soxR* regions of tigecycline-resistant isolates are identical to those of the susceptible isolates, and the sequences also align with the reference sequence of tigecycline-susceptible isolate *K*. *pneumoniae* subsp. *pneumoniae* MGH 78578 (GenBank accession no. CP000647) [15].

Mutations in the *acrR* gene were observed in 14 isolates (Table 3), and 12 isolates have IS*5* insertion element into the nucleotide position 276–277 of the gene and two isolates harboured point mutations. The expression level of the *acrB* gene in the isolates harboring mutant *acrR* genes were higher than the control strain K134 which with a wild-type *acrR* gene, but not significant.

Mutations in the *ramR* gene were observed in three resistant isolates (Y17, H65, S21). One isolate (S21) harboured a point mutation leading to a premature stop codon, which resulted in a predicted truncated RamR protein that was most likely non-functional, and two isolates (Y17, H65) harboured point mutations leading to amino acid exchanges in the coding region of *ramR* (Table 3). Overexpressing of *ramA* could be due to the mutations of *ramR* in these strains.

### *ramR* mutations contribute to tigecycline resistance in clinically isolated *Klebsiella pneumoniae*

Transformation of S21 with wild-type *ramR*_K134_ (S21/*ramR*_K134_) lowered the MIC for tigecycline from 8 mg/L to 1 mg/L. The influence of *ramR* mutations on the transcript expression levels of *ramA* and *acrB* in S21 was analysed by real-time PCR. Transformation of wild-type *ramR* (from K134) into S21 (S21/*ramR*_K134_) resulted in strongly repressed *ramA* expression (14.20-fold), and *acrB* expression was also downregulated (4.73-fold) (Table 6). No change was noted when transformed with the empty pCR-BluntII -TOPO vector (S21/ pCR-BluntII -TOPO). These data indicate that *ramR* mutations via *ramA* activation subsequently resulted in the up-regulation of efflux pump *acrAB* contribute to tigecycline resistance in *K*. *pneumoniae* clinical isolates.

**Table 6:** Tigecycline MIC and relative expressions of *ramA* and *acrB* when complemented with wild-type *ramR* in S21.

Isolates	MIC (mg/L) ^b^	*ramR* mutations	Relative expression ^a^	
			*ramA*	*acrB*
S21	8	Q122Stop	13.77±2.90	3.97±0.49
S21/*ramR*_K134_	1		0.97±0.22	0.84±0.14
S21/ pCR-BluntⅡ-TOPO	8		12.91±1.77	3.55±0.41
K134	0.25		1	1

^a^ Relative expression compared with K134 (expression = 1). Results are means of 3 runs ± standard deviation.

^b^ MIC of tigecycline.

## Discussion

KPC-producing *K*. *pneumoniae* isolates have emerged as important pathogens of nosocomial infections. These strains often show resistance to almost all antibiotics, and their worldwide spread usually causes a great threat to public health. Tigecycline is one of the antibiotics recommended for severe infections caused by KPC-producing *K*. *pneumoniae* [20]. In This study, we identified the susceptibility profile of tigecycline in KPC-producing *K*. *pneumoniae* in China. The MIC range and MIC_90_ in these isolates are identical to previous findings in the USA in which the activity of tigecycline was determined in multidrug-resistant *K*. *pneumoniae* [6]. According to US FDA criteria, the resistance rate in these isolates was 4.7% (MIC ≥8 mg/L), and the susceptibility rate was 88.8% (MIC≤2 mg/L). It appears that tigecycline retains good activity against KPC-producing *K*. *pneumoniae* in *vitro*.

Tigecycline resistance occurring in *K*. *pneumoniae* during therapy has recently been reported in many cases [21–24]. The mechanism of resistance is not yet clear. However, it is becoming apparent that the development of resistance to tigecycline is rather complicated, and more than one mechanism may be involved. In This study, 24 isolates belong to 9 clonal groups show resistance to tigecycline. Exposure of these isolates to the efflux pump inhibitor NMP resulted in an obvious decrease in the MICs of tigecycline and restored susceptibility to tigecycline in 91.7% of the isolates. These data suggests that efflux pumps are involved in decreased tigecycline susceptibility. However, the effects of PAβN and CCCP were not significant. Kern WV et al. deem that different antibiotics may have different binding sites on the pump with which the EPIs might interfere in a variable manner [25]. The different effect of the three EPIs on tigecycline MICs might be due to the different action mode of the EPIs and the particular binding sites of tigecycline. AcrAB-TolC, an RND-type efflux pump, has been linked to the non-susceptibility of tigecycline in a variety of *Enterobacteriaceae* [4, 8, 15, 16, 26]. Our results support the hypothesis that increased expression of the AcrAB pump is associated with increased MICs of tigecycline in *K*. *pneumoniae*. However, in two strains (K22, K83), the expression level of *acrB* was relatively low, suggesting that efflux pumps other than AcrAB may take effect in these strains. We also examined the expression level of *oqxB* in This study, but the role of OqxAB pump in tigecycline resistance was uncertain.

Transcriptional activators RamA, MarA, SoxS, and RarA have been linked to efflux pump-mediated resistance to tigecycline [5, 11, 12, 16]. However, the difference of *marA*, *soxS*, and *rarA* expression between tigecycline resistant and susceptible isolates were not significant, which indicating *marA*, *soxS*, and *rarA* may not in the domination position in tigecycline resistance of *K*. *pneumoniae*.

Mutations in the *acrR* gene were observed in some isolates. The expression level of the *acrB* gene in the isolates harboring mutant *acrR* genes were higher than those with a wild-type *acrR* gene, but not significant. The *acrR* gene mutations may partly contribute to AcrAB pump-mediated tigecycline resistance in *K*. *pneumoniae*. Mutations in the *ramR* gene were observed in three tigecycline resistant isolates (Y17, H65, S21). The high expression of *acrB* in isolate S21 could be due to mutations in *ramR* which led to the up-regulation of *ramA*. Moreover, isolates Y17 and H65 both have *acrR* mutation and *ramR* mutation, and the two mutations may together contribute to the overexpression of *acrB*.

For the one tigecycline-resistant strain (K23) that is insensitive to efflux pump inhibitors and had low expression of all genes examined in This study, it is likely that other mechanisms are involved in the development of tigecycline resistance.

## Supporting Information

S1 Fig Phylogenetic clone analysis of 24 tigecycline MIC = 1 mg/L isolates. These isolates were divided into 13 clonal groups.
(TIF)

S2 Fig Phylogenetic clone analysis of seven tigecycline MIC = 0.25 mg/L isolates. These isolates were divided into 7 clonal groups.
(TIF)
