# Supplementary figures and images for: Tigecycline Susceptibility and the Role of Efflux Pumps in Tigecycline Resistance in KPC-Producing Klebsiella pneumoniae
Source: PLoS One. 2015 Mar 3;10(3):e0119064. doi: 10.1371/journal.pone.0119064 (PMC4348519; doi:10.1371/journal.pone.0119064)

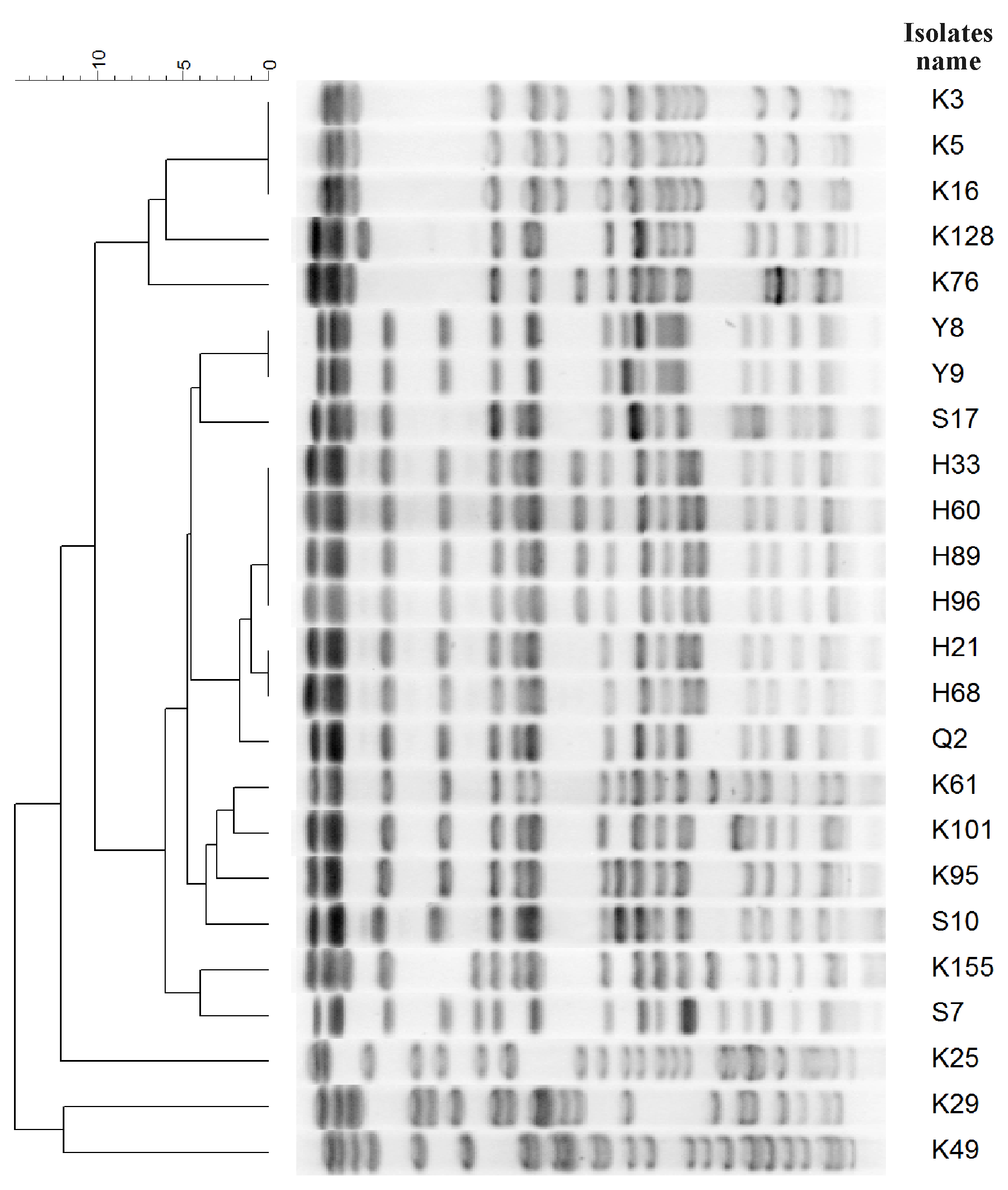

Supplement: S1 Fig — These isolates were divided into 13 clonal groups. (TIF) [file pone.0119064.s001.tif]

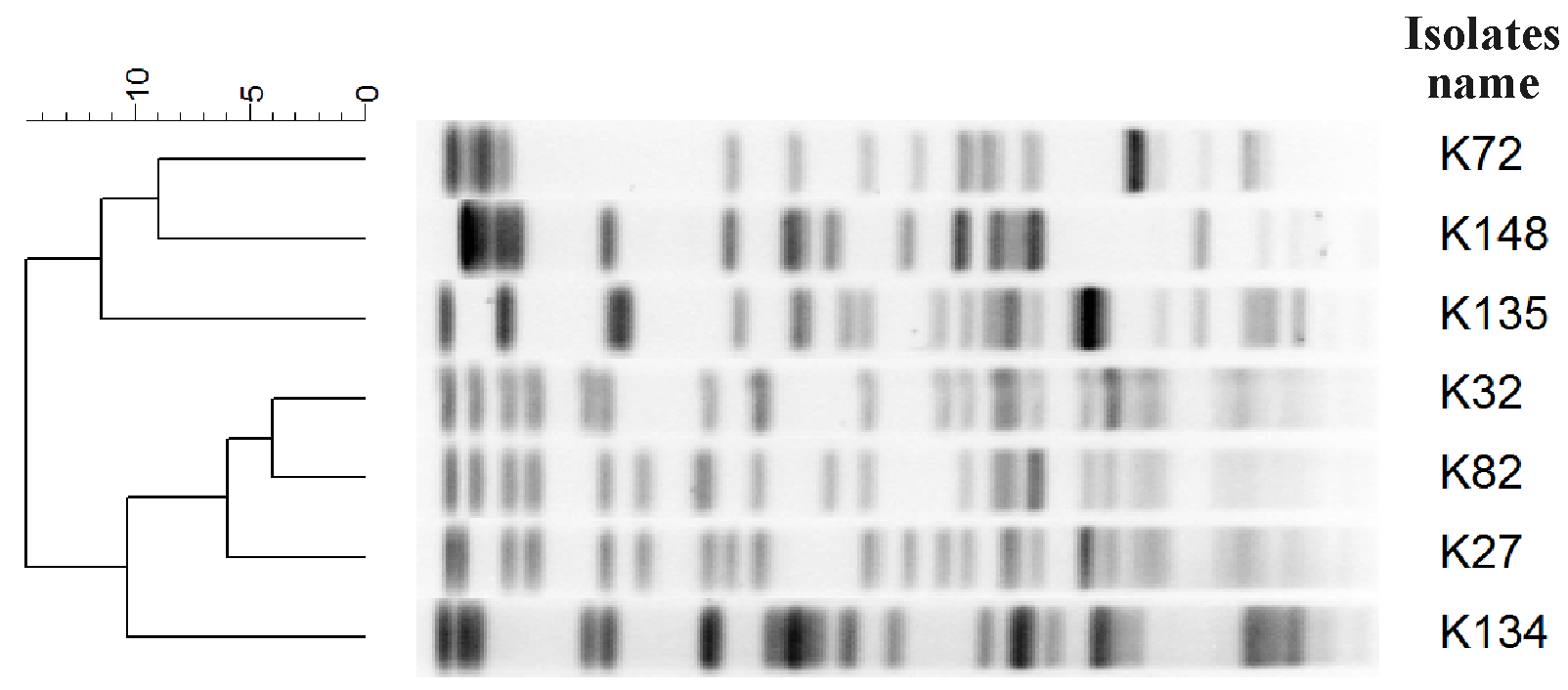

Supplement: S2 Fig — These isolates were divided into 7 clonal groups. (TIF) [file pone.0119064.s002.tif]
